# Esophageal metastasis of renal cell carcinoma resected by endoscopic submucosal dissection: a case report

**DOI:** 10.1186/s12876-021-01925-z

**Published:** 2021-09-20

**Authors:** Ken Ohnita, Shuntaro Higashi, Satoshi Hirai, Ai Kuwahara, Kana Kakigao, Suguru Nakashiki, Kenichiro Inoue, Hirokazu Kurohama, Masahiro Nakashima, Kazuhiko Nakao

**Affiliations:** 1 Department of Gastroenterology and Hepatology, Shunkaikai Inoue Hospital, 6-12 Takara-machi, Nagasaki, Japan; 2 Department of Gastroenterology and Hepatology, Graduate School of Biomedical Sciences, Nagasaki University, 1-7-1 Sakamoto, Nagasaki, Japan; 3 Department of Tumor and Diagnostic Pathology, Atomic Bomb Disease Institute, Nagasaki University, 1-12-4 Sakamoto, Nagasaki, Japan

**Keywords:** Renal cell carcinoma, Esophageal metastasis, Endoscopic submucosal dissection

## Abstract

**Background:**

Esophageal metastasis of renal cell carcinoma (RCC) is extremely rare. We have described herein a case of a 59-year-old man with esophageal metastasis of RCC that was endoscopically resected.

**Case presentation:**

The case was a 59-year-old man who had undergone left nephrectomy for renal clear cell carcinoma 17 years ago and splenectomy for splenic metastasis 3 years ago. Esophagogastroduodenoscopy (EGD) performed 9 years ago revealed a small reddish elevated lesion with a smooth surface in the middle esophagus; this lesion increased in size 4 years ago. However, no biopsy was performed. The lesion continued to grow in size and was found to have become nodular during the present observation. Biopsy revealed clear cell carcinoma. Endoscopic ultrasound (EUS) revealed that the lesion had not invaded the submucosa, and contrast-enhanced computed tomography did not reveal any other metastasis. The lesion was successfully removed en bloc via endoscopic submucosal dissection (ESD). Pathologically, the tumor was detected in the subepithelium with focal infiltration of the muscularis mucosa. It consisted of monotonous cells with small nuclei and a clear cytoplasm. Immunohistological findings indicated that the tumor was a metastasis of RCC. The lateral and vertical margins were noted to be free.

**Conclusions:**

We have presented herein a case of esophageal metastasis of RCC that had progressed over 9 years and was then resected en bloc through endoscopic submucosal dissection.

## Background

Esophageal metastasis of RCC is extremely rare. Most cases of this disease are detected when the tumor attains a large size and rarely when it is small. We have described here a case of a 59-year-old man with esophageal metastasis of RCC that had progressed over 9 years and then finally removed en bloc by ESD.

## Case presentation

**Fig. 1:**
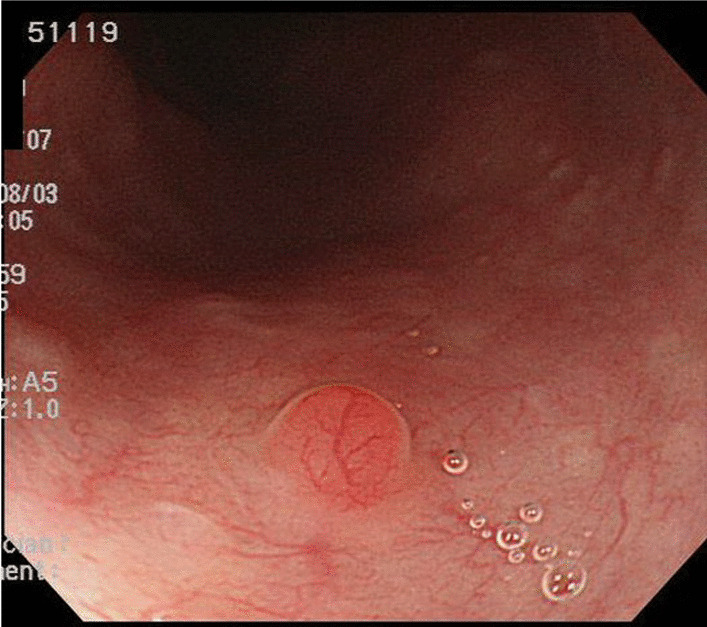
EGD disclosed a small reddish elevated lesion whose surface was smooth at the middle esophagus

**Fig. 2:**
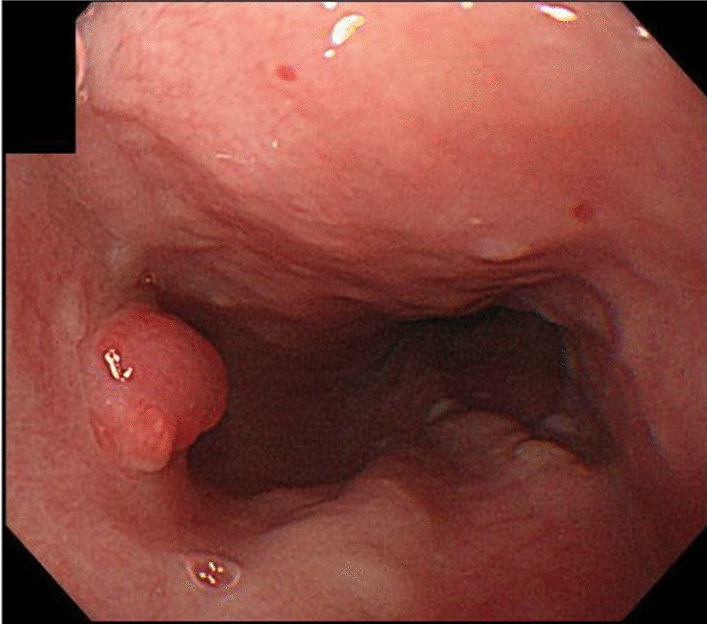
The lesion increased in size 4 years ago

**Fig. 3:**
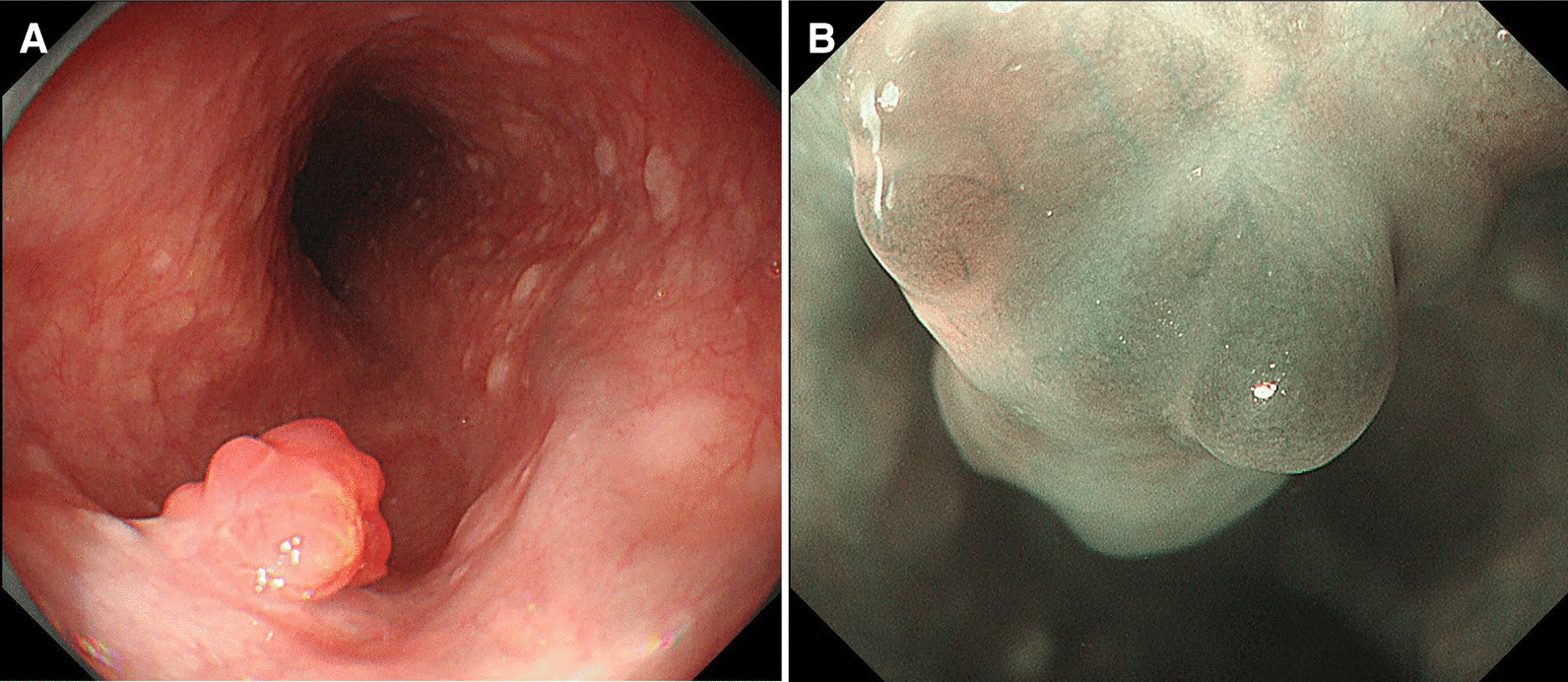
**A** The lesion further increased in size and was found to have become nodular during the present observation. **B** Magnifying narrow band imaging endoscopy revealed a dark-green tumor, but no intraepithelial papillary capillary loop abnormality was detected

**Fig. 4:**
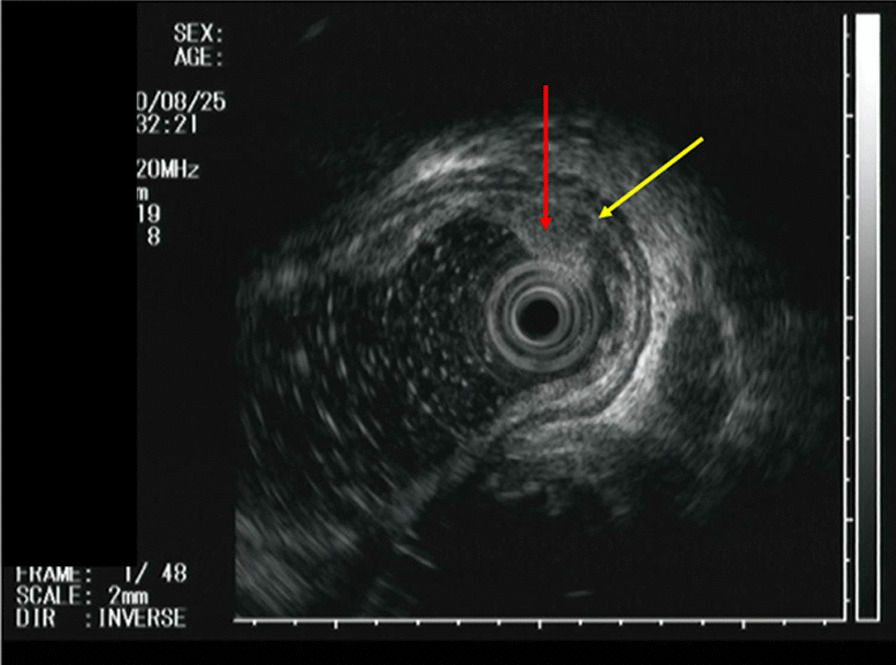
EUS showing that the lesion (red arrow) did not invade to the submucosa (yellow arrow)

**Fig. 5:**
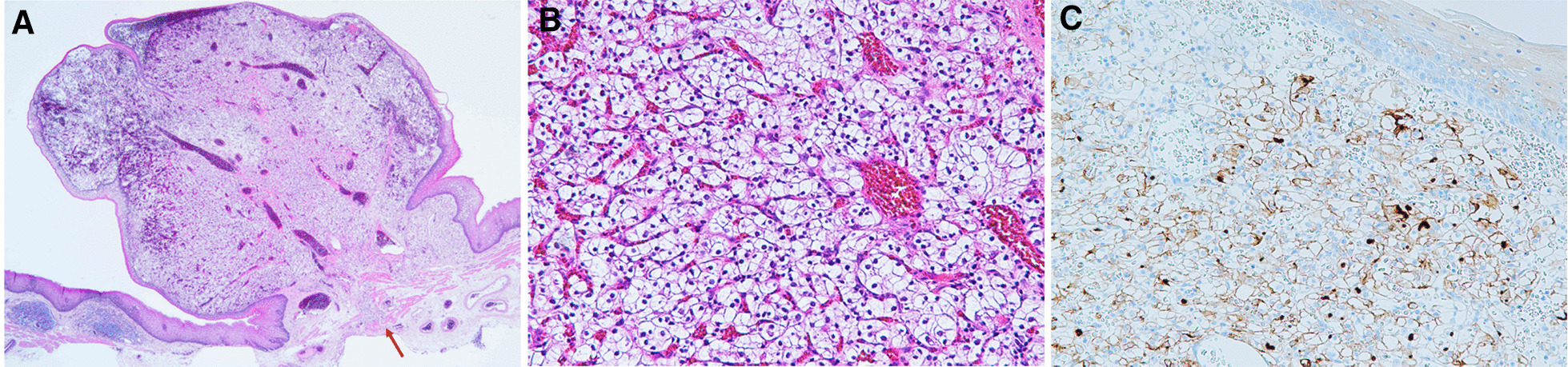
**A** The tumor (5 × 4 mm) present in the subepithelium with focal infiltration to the muscularis mucosa (arrow) (2 × , hematoxylin–eosin stain). **B** The tumor comprised monotonous cells with small nuclei and a clear cytoplasm (20 × , hematoxylin–eosin stain). **C** The tumor cells were immunohistochemically positive for CD10

The case was a 59-year-old man who had undergone left nephrectomy for renal clear cell carcinoma 17 years ago and splenectomy for splenic metastasis 3 years ago. He then underwent ESD for early gastric cancer 9 years ago. At that time, EGD revealed a small reddish elevated lesion with a smooth surface in the middle esophagus (Fig. 1), and the lesion had increased in size 4 years ago during surveillance scope (Fig. 2). However, no biopsy was performed because the endoscopist assumed the lesion to be a benign polyp. We performed EGD during the next follow-up to asses it. The lesion continued to increase in size to approximately 10 mm and was found to have become nodular (Fig. 3A). Magnifying narrow band imaging endoscopy revealed a dark-green tumor, although no intraepithelial papillary capillary loop abnormality was detected (Fig. 3B). We suspected that it was a metastasis from the RCC because the polyp was red in color, looked like a submucosal tumor, and had increased in size. Biopsy revealed clear cell carcinoma. EUS demonstrated that the lesion had not invaded the submucosa (Fig. 4), and contrast-enhanced computed tomography did not reveal any other metastasis. The lesion was then successfully removed en bloc by ESD. Pathologically, the tumor (5 × 4 mm) was observed in the subepithelium with a focal infiltration of the muscularis mucosa (Fig. 5A). It comprised monotonous cells with small nuclei and a clear cytoplasm (Fig. 5B) and was found to be immunohistochemically positive for AE1/AE3, CD10 (Fig. 5C), and vimentin, but negative for chromogranin A, synaptophysin, and CD56. The tumor was considered to be a metastasis of RCC. The lateral and vertical margins were noted to be free. We successfully resected the lesion endoscopically and followed it up with observation.

## Discussion and conclusion

Metastatic esophageal cancer is a considerably rare occurrence. Two mechanisms are believed to be involved in metastasis from other organs to the esophagus: (i) the first one is through the direct invasion of tissues, such as the lung, trachea/bronchus, thyroid, larynx, hypopharynx, and stomach tissues and (ii) the second one is through hematogenous/lymphogenous invasion of the distant organs, such as the uterus or liver [1]. RCC has a tendency to hematogenously metastasize to the lung, bone, or liver. However, metastasis to the esophagus is extremely rare [1]. Mizobuchi et al. reviewed an autopsy data of 1835 patients who died of cancer. They reported that 112 (6.1%) cases had metastasis to the esophagus and detected common primary tumor of the lung in 51 patients, of the breast in 14 patients, and of the stomach in 13 patients [2]. A PubMed search with the keywords “renal cell carcinoma,” “renal cancer,” and “esophageal metastasis” retrieved only seven studies on esophageal metastasis of RCC [1, 3–8]. The endoscopic features of esophageal metastasis from RCC were reported with a pedunculated polypoid tumor [1], fragile mass [6], and reddish tumor with ulcer [7]. Most cases had presented symptoms such as dysphagia, melena, and hematemesis. Our case was asymptomatic; thus, we noted that the lesion size was small. Patients treated with complete metastasectomy showed better survival and symptom control than those treated with either incomplete or no metastasectomy in a past study [9]. According to the National Comprehensive Cancer Network Guidelines (version 4, 2018), surgical treatment is indicated when the primary tumor site is the kidney and the patient has a resectable solitary metastasis [10]. Monteros-Sanchez et al. [4] and Izumo et al. [1] underwent esophagectomy for metastasis from RCC. If the tumor is unresectable, multidisciplinary treatments including radiation and chemotherapy with molecular-targeting therapy agents, such as tyrosine-kinase inhibitors, are indicated [6]. As the metastatic lesion appeared to be intramucosal from the endoscopic ultrasound, we decided to perform ESD with adequate margin. Padda et al. diagnosed the metastatic RCC by biopsy and accordingly performed ESD [8]. However, they did not detect any malignant tumor in the ESD specimen. The metastatic lesion may have been removed by biopsy. Therefore, to our knowledge, our patient represents the first case of successful removal by ESD. We could thus conduct a complete resection because the lesion remained in the muscularis mucosa and was negative for lateral and vertical margin. RCC is a slow growing tumor. In our case, esophageal metastasis occurred 8 years after left nephrectomy. Unfortunately, we could not diagnosis the metastasis because we did not perform biopsy at that time. However, it is a valuable case that showed progression over 9 years. Based on our experience, cases of RCC metastasis to the esophagus may increase in the future considering that RCC is a slow growing tumor, which necessitates development in endoscopic diagnosis.

In conclusion, we presented a case of esophageal metastasis of RCC that progressed over 9 years and was successfully resected en bloc by ESD.

## Data Availability

The datasets used in the current study are available from the corresponding author on reasonable request.

## Abbreviations

RCC: Renal cell carcinoma
EGD: Esophagogastroduodenoscopy
ESD: Endoscopic submucosal dissection
EUS: Endoscopic ultrasound

## References

[1] Izumo W, Ota M, Narumiya K (2015). Esophageal metastasis of renal cancer 10 years after nephrectomy. Esophagus.

[2] Mizobuchi S, Tachimori Y, Kato H (1997). Metastatic esophageal tumors from distant primary lesions: report of three esophagectomies and study of 1835 autopsy cases. Jpn J Clin Oncol.

[3] Trentino P, Rapacchietta S, Silvestri F (1997). Esophageal metastasis from clear cell carcinoma of the kidney. Am J Gastroenterol.

[4] de los Monteros-Sanchez AE, Medina-Franco H, Arista-Nasr J (2004). Resection of an esophageaal metastasis from a renal cell carcinoma. Hepatogastroenterology.

[5] Cabezas-Camarero S, Puente J, Manzano A (2015). Renal cell cancer metastases to esophagus and stomach successfully treated with radiotherapy and pazopanib. Anticancer Drugs.

[6] Ali S, Atiquzzaman B, Krall K (2018). Not your usual suspect: clear cell renal cell carcinoma presenting as ulcerative esophagitis. Cureus.

[7] Thomas AS, Schwartz MR, Neshatian L (2018). A rare cause of an upper gastrointestinal bleed. Gastroenterology.

[8] Padda MS, Si WM (2019). Rare presentation of renal cell cancer as dysphagia: a case report. J Med Case Rep.

[9] Dabestani S, Marconi L, Hofmann F (2014). Local treatments for metastases of renal cell carcinoma: a systematic review. Lancet Oncol.

[10] https://www2.tri-kobe.org/nccn/guideline/urological/english/kidney.pdf

